# Increased nuclear stiffness via FAK-ERK1/2 signaling is necessary for synthetic mechano-growth factor E peptide-induced tenocyte migration

**DOI:** 10.1038/srep18809

**Published:** 2016-01-08

**Authors:** Bingyu Zhang, Qing Luo, Zhen Chen, Yisong Shi, Yang Ju, Li Yang, Guanbin Song

**Affiliations:** 1 Key Laboratory of Biorheological Science and Technology, Ministry of Education, College of Bioengineering, Chongqing University, Chongqing 400044, China; 2 Department of Mechanical Science and Engineering, Nagoya University, Nagoya 464-8603, Japan

## Abstract

We have previously reported that a synthetic mechano-growth factor (MGF) C-terminal E-domain with 25 amino acids (MGF-C25E) promotes rat tenocyte migration through the FAK-ERK1/2 signaling pathway. However, the role of the nucleus in MGF-C25E-promoted tenocyte migration and the molecular mechanisms involved remain unclear. In this study, we demonstrate that MGF-C25E increases the Young’s modulus of tenocytes through the FAK-ERK1/2 signaling pathway. This increase is not accompanied by an obvious change in the expression of Lamin A/C but is accompanied by significant chromatin condensation, indicating that MGF-C25E-induced chromatin condensation may contribute to the increased nuclear stiffness. Moreover, DNA methylation is observed in MGF-C25E-treated tenocytes. Inhibition of DNA methylation suppresses the elevation in chromatin condensation, in nuclear stiffness, and in tenocyte migration induced by MGF-C25E. The inhibition of the focal adhesion kinase (FAK) or extracellular signal regulated kinase 1/2 (ERK1/2) signals represses MGF-C25E-promoted DNA methylation. It also abolishes chromatin condensation, nuclear stiffness, and cell migration. Taken together, our results suggest that MGF-C25E promotes tenocyte migration by increasing nuclear stiffness via the FAK-ERK1/2 signaling pathway. This provides strong evidence for the role of nuclear mechanics in tenocyte migration and new insight into the molecular mechanisms of MGF-promoted tenocyte migration.

A tendon is a tough band of fibrous connective tissue that connects muscle to bone. This allows the transmission of forces generated by muscle to bone, which results in joint movement. However, inappropriate or excessively repetitive stretches of physical training often result in overuse tendon injuries. Poor blood supply to the tendon and a modest metabolic rate make tendon injuries difficult to treat. This often results in slow/incomplete healing, long-term functional disability and pain after injury^1^,^2^. How to manage the damaged tendon is still one of the most challenging problems in orthopedics.

Biological augmentation of tendon healing with growth factors has shown promising preclinical results. Mechano-growth factor (MGF) is an alternative splice variant of the insulin-like growth factor-1 (*igf-1*) gene, and it takes its name from the first study that linked this variant to mechanical stimuli in muscle^3^. In humans and rodents, the *igf-1* gene spans more than 90 kb of chromosomal DNA and contains six exons. Alternative splicing of IGF-1 pre-mRNA can produce multiple mRNA species. The *igf-1* gene generates three isoforms in humans, namely, IGF-1 Ea, IGF-1 Eb and IGF-1 Ec^4^. However, *igf-1* generates only two isoforms in rats, namely, IGF-1 Ea and IGF-1 Eb^5^. Rodent IGF-1 Eb and human IGF-1 Ec are named MGF (Fig. 1A). In rats, the 52-bp insertion (49-bp in human) in exon 5 causes a frame-shift that generates a variant containing a different C-terminal E-domain with 25 amino acids (24 amino acids in human), which is called MGF-C25E (MGF-C24E in human) (Fig. 1B)^4^,^5^. Because only the alternate E domains are different in all splice variants, research on MGF usually focuses on the role of the MGF E domain^6^,^7^. MGF has been shown to activate satellite cells in muscles, resulting in hypertrophy or regeneration. It has also been shown to function as a neuroprotectant in brain ischemia^7^,^8^,^9^. Moreover, previous studies demonstrated that MGF promotes bone-defect healing and induces more blood vessels in bone regeneration around the defective areas^6^,^10^. However, it remains unclear whether MGF has the potential to accelerate tendon repair.

The important role of cell movement in multiple biological processes such as embryonic development, immune response, wound healing and tissue renewal makes it one of the most fundamental cellular activities. In the early stages of tendon healing, tenocytes gradually move to the wound and proliferate while secreting collagens and glycoproteins for regeneration^11^. Cell movement is a complex process requiring motor proteins and coordinated structural changes in multiple cellular components^12^,^13^. Research about cell movement has predominantly focused on the cytoskeleton, adhesion complexes and signaling molecules. Recently it has been found that nuclear shape, size, stiffness and plasticity may play an important role in cell movement^14^,^15^.

Cell movement can lead to nuclear changes at three levels: transcription of specific genes, the shape of the nucleus and the localization of the nucleus within the cell^16^. Transcription of genes encoding proteins, such as plasma membrane receptors, focal adhesion proteins and cytoskeletal proteins, are involved in the migration process^17^,^18^. Generally, cell movement can lead to translocation of the cell body across two-dimensional (2D) surfaces, through basement membranes or three-dimensional (3D) interstitial tissues^19^. During migration through 3D tissues, the stiffness and density of the surrounding extracellular matrix (ECM) or the cells themselves present a physical challenge to the moving cell body. The cell movement through 3D tissues is strongly dependent on the deformation of the cells. The nucleus is the largest and stiffest organelle, so its size and relative stiffness can pose a major obstacle for cell migration through narrow openings in the ECM or inside layers of cells. However, cell movement is often, but not always, associated with changes in nuclear shape. In 2D surfaces, the cells often spread out significantly, resulting in more disk-shaped nuclei and a height of only a few micrometers^20^. Cell migration is associated with cytoskeleton-mediated relocation of the nucleus within the cells, and an increase in nuclear stiffness can enhance the ability of cytoskeletal elements to relocate the nucleus inside the cell^16^.

In our previous studies, we demonstrated that MGF-C25E promotes rat tenocyte migration and invasion via the FAK-ERK1/2 signaling pathway^21^,^22^. However, little is known about the role of the nucleus in MGF-C25E-promoted tenocyte migration and the molecular mechanisms involved. In this study, we aimed to explore the effect of MGF-C25E on nuclear mechanics of tenocytes and its roles and mechanisms in the process of MGF-C25E-promoted tenocyte migration.

## Results

### MGF-C25E increases stiffness of the nucleus via the FAK-ERK1/2 signaling pathway

**Figure 2:**
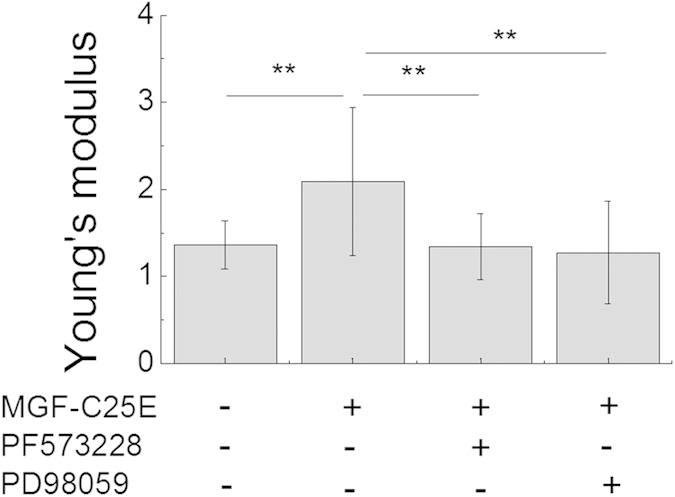
Atomic force microscopy (AFM) analysis of tenocyte nuclear stiffness as evaluated by Young’s modulus. The nuclei of tenocytes exposed to MGF-C25E (30 ng/mL) exhibited a higher Young’s modulus than those of control cells (non-MGF-C25E-treated cells), whereas pretreatment with FAK inhibitor PF573228 (10 μM) or ERK1/2 inhibitor PD98059 (50 μM) for 30 min prevented the increase of Young’s modulus in tenocytes induced by MGF-C25E. The data are expressed as the means ± SD; n = 20 in each group; **p < 0.01.

Nuclei were isolated from MGF-C25E-treated tenocytes with or without 10 μM PF573228 (a selective inhibitor of FAK) or 50 μM PD98059 (a specific inhibitor of MEK). The Young’s modulus of the nucleus was assessed by atomic force microscopy (AFM). The AFM results showed that the Young’s modulus of tenocyte nuclei exposed to MGF-C25E was apparently higher than that of the control, showing that the nucleus was stiffer after exposure to MGF-C25E. Moreover, the inhibition of the FAK or ERK1/2 signal suppressed the MGF-C25E-induced increase in the Young’s modulus (Fig. 2). These results demonstrate that MGF-C25E increases the stiffness of tenocyte nuclei via the FAK-ERK1/2 signaling pathway.

### MGF-C25E has no effect on Lamin A/C expression

**Figure 3:**
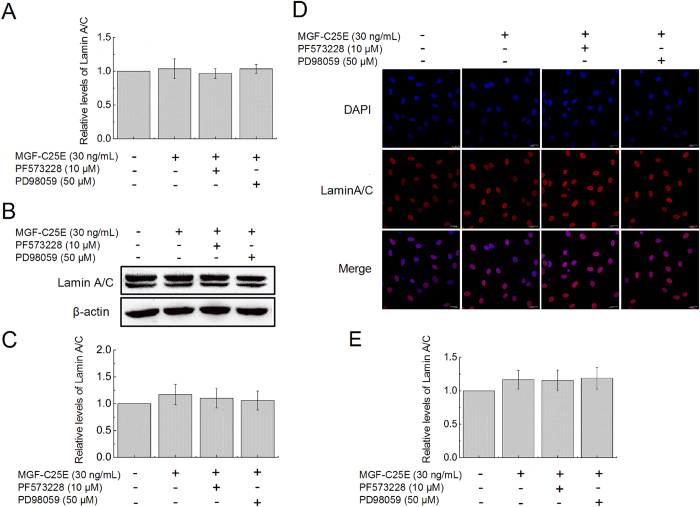
Effect of MGF-C25E on Lamin A/C expression in tenocytes. (**A**) qRT-PCR was used to detect the gene expression of Lamin A/C in MGF-C25E-treated (30 ng/mL) tenocytes with or without PF573228 (10 μM) or PD98059 (50 μM). The protein expression of Lamin A/C was detected by western blot (**B,C**) and immunostaining (**D,E**). Bars = 20 μm. The data are expressed as the means ± SD; n = 3 in each group.

To determine whether the MGF-C25E promotes Lamin A/C expression, which contributes to regulation of nuclear stiffness and the involved signaling molecules, tenocytes were exposed to MGF-C25E for 24 h with or without FAK inhibitor or ERK 1/2 inhibitor. The expression of Lamin A/C was detected by qRT-PCR, western blot, and immunostaining. We found that MGF-C25E had no significant effect on Lamin A/C expression in tenocytes both at the mRNA (Fig. 3A) and protein levels (Fig. 3B–E). Blockage of the FAK-ERK1/2 signaling pathway did not affect the expression of Lamin A/C (Fig. 3). These results suggest that Lamin A/C does not contribute to the MGF-C25E-induced increase in tenocyte nuclear stiffness.

### MGF-C25E increases chromatin condensation via the FAK-ERK1/2 signaling pathway

**Figure 4:**
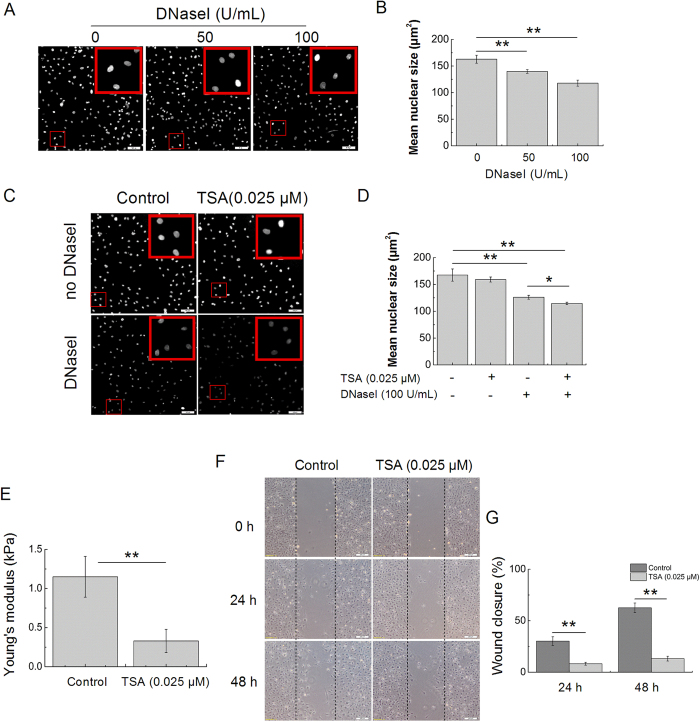
Chromatin decondensation reduces nuclear stiffness and cell migration. (**A**) *In situ* DNaseI-sensitivity assay. Photomicrographs of DAPI-stained nuclei after 20 minutes of digestion with the different concentrations of DNaseI. Bars = 100 μm. (**B**) Quantitative analysis of the mean size of nuclei after DNaseI as shown in **(A)**. n = 3 in each group. (**C**) Validation of the *in situ* DNaseI-sensitivity assay: decondensation of chromatin enhances the rate of digestion. Photomicrographs show that the nuclear sizes of the TSA-treated cells are significantly decreased when compared to those of the control cells. Bars = 100 μm. (**D**) Quantitative analysis of the mean size of nuclei after DNaseI as shown in **(C)**. n = 3 in each group. (**E**) The nuclei of tenocytes exposed to TSA (0.025 μM) are less stiff than those of the control cells (non-TSA-treated). n = 15 in each group. (**F**) Images of the migration of tenocytes treated with TSA, as evaluated through a scratch wound assay. Bars = 200 μm. (**G**) Percentages of wound closure obtained for tenocytes treated with TSA. n = 3 in each group. The data are expressed as the means ± SD; *p < 0.05 and **p < 0.01.

Chromatin condensation is another contributor to the stiffness of the nucleus. To determine whether MGF-C25E affects chromatin condensation, we adapted the well-established chromatin DNaseI sensitivity assay to test chromatin condensation. Measurement of the relative nuclear size is a reliable indicator of DNaseI sensitivity and can therefore be used to assess the relative levels of chromatin condensation^23^,^24^. As shown in Fig. 4A, the mean size of the nuclei without DNaseI treatment was approximately 162 μm^2^. When the cells were treated with 50 U/mL DNaseI, the mean size of the nuclei was decreased to approximately 140 μm^2^. An increase in the DNaseI concentration (100 U/mL) further reduced the nuclear size, and the mean size of nuclei was only approximately 118 μm^2^ (Fig. 4A,B).

To further verify that the *in situ* DNaseI-sensitivity assay was indeed adequate for assessing the relative levels of chromatin condensation, we compared the DNaseI sensitivity of cells treated with the histone deacetylase (HDAC) inhibitor trichostatin A (TSA). TSA is known to cause decondensation of chromatin^25^. As shown in Fig. 4C,D, the size of the nuclei in TSA-treated cells significantly decreased when compared to that of the control cells. The accelerated decrease in the nuclear size of TSA-treated cells indicated that DNaseI digested the chromatin more rapidly. Moreover, TSA significantly decreased the Young’s modulus of tenocyte nuclei, resulting in softer nuclei in comparison with the non-TSA-treated nuclei (Fig. 4E). Treatment with TSA also caused notably lower wound closure of tenocytes compared with the control (without TSA treatment) (Fig. 4F,G). These results indicate that TSA-induced decondensation of chromatin results in reduced nuclear stiffness and decreased migration of tenocytes.

**Figure 5:**
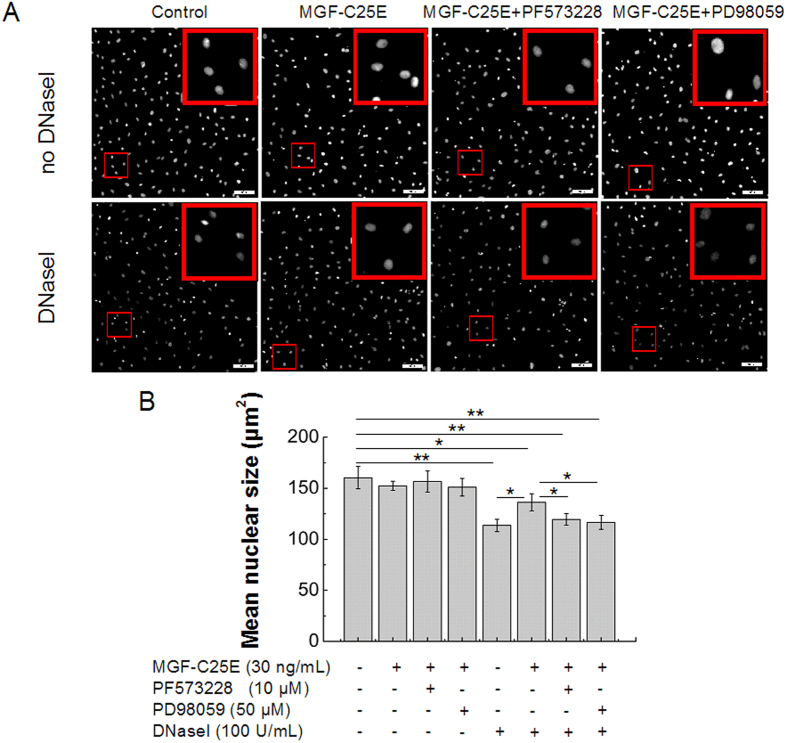
Analysis of chromatin condensation state by *in situ* DNase I-sensitivity assay. (**A**) Validation of the *in situ* DNaseI-sensitivity assay: MGF-C25E reduces the rate of digestion. Photomicrographs show that the nuclear sizes of the MGF-C25E-treated cells are significantly increased when compared to those of the control cells. The inhibition of the FAK or ERK1/2 signals repressed the MGF-C25E-induced increase in the nuclear size. Bars = 100 μm. (**B**) Quantitative analysis of the mean size of nuclei after DNaseI as shown in A. Data are expressed as the means ± SD; n = 3 in each group; *p < 0.05 and **p < 0.01.

Next, the *in situ* DNaseI-sensitivity assay was used to compare the chromatin condensation state in MGF-C25E-treated tenoctyes with or without PF573228 or PD98059. As shown in Fig. 5, the mean size of the nuclei of MGF-C25E-treated cells significantly increased compared to that of the control cells. The inhibition of the FAK or ERK1/2 signal repressed the MGF-C25E-induced increase in the nuclear size (Fig. 5A, B). These results demonstrate that MGF-C25E promotes chromatin condensation through the FAK-ERK1/2 signaling pathway in tenocytes.

### MGF-C25E elevates DNA methylation via the FAK-ERK1/2 signaling pathway

**Figure 6:**
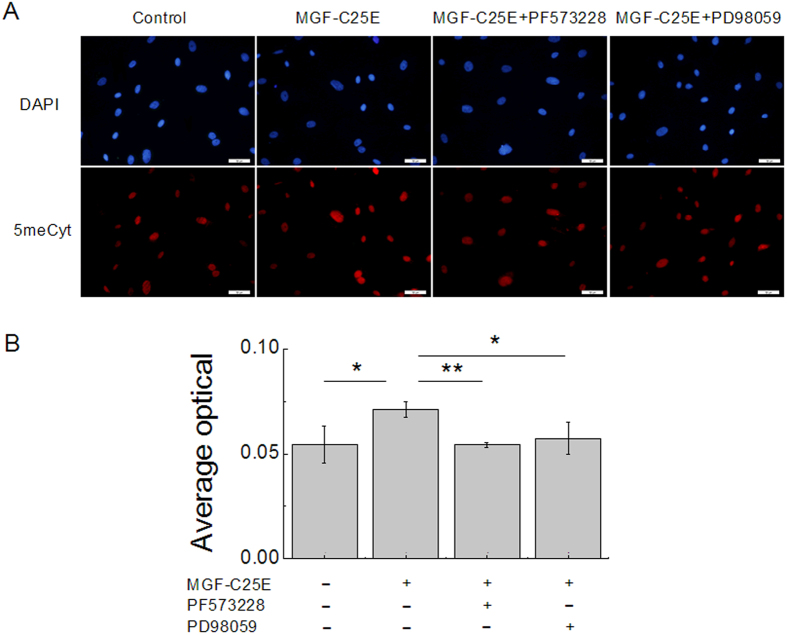
MGF-C25E elevates DNA methylation via the FAK-ERK1/2 signaling pathway. (**A**) Immunofluoresence staining of 5-methyl-Cytidine (5meCyt, red) and the nucleus (blue) in MGF-C25E-treated tenocytes with or without PF573228 or PD98059. Bars = 50 μm. (**B**) The representative bands show the average optical density of 5meCyt. The data are expressed as the means ± SD; n = 3 in each group; *p < 0.05 and **p < 0.01.

DNA methylation has been implicated in chromatin condensation and nuclear organization. To reveal the possible role of DNA methylation in MGF-C25E-promoted chromatin condensation, tenocytes were exposed to MGF-C25E for 24 h with or without PF573228 or PD98059, and the levels of DNA methylation were detected by immunostaining. As shown in Fig. 6, the DNA methylation level increased significantly in MGF-C25E-treated tenocytes. The inhibition of the FAK or ERK1/2 signals suppressed MGF-C25E-induced DNA methylation (Fig. 6A,B), suggesting that MGF-C25E induces DNA methylation via the FAK-ERK1/2 signaling pathway in tenocytes.

### DNA methylation increases chromatin condensation, nuclear stiffness, and tenocyte migration

**Figure 7:**
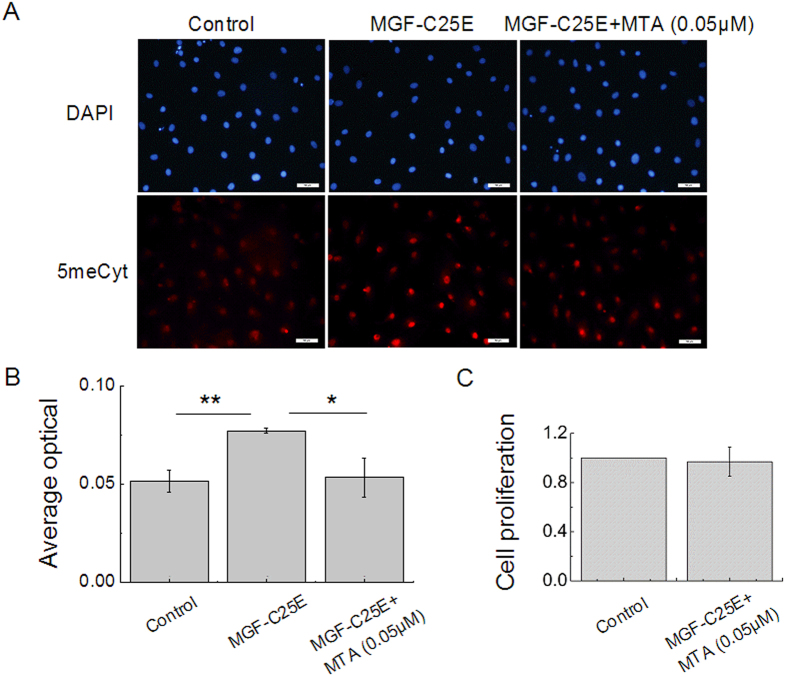
Effect of DNA methylation inhibitor (MTA) on MGF-C25E-promoted DNA methylation. (**A**) The relative levels of 5meCyt. Tenocytes were exposed to MGF-C25E (30 ng/mL) in the absence or the presence of MTA (0.05 μM) for 24 h, after which the methylation level was detected by immunostaining. Bars = 50 μm. (**B**) Quantitative analysis of the relative levels of 5meCyte in tenocytes. (**C**) The MTT assay shows that MTA at 0.05 μM has no effect on tenocyte viability within 24 h. The data are expressed as the means ± SD; n = 3 in each group; *p < 0.05 and **p < 0.01.

**Figure 8:**
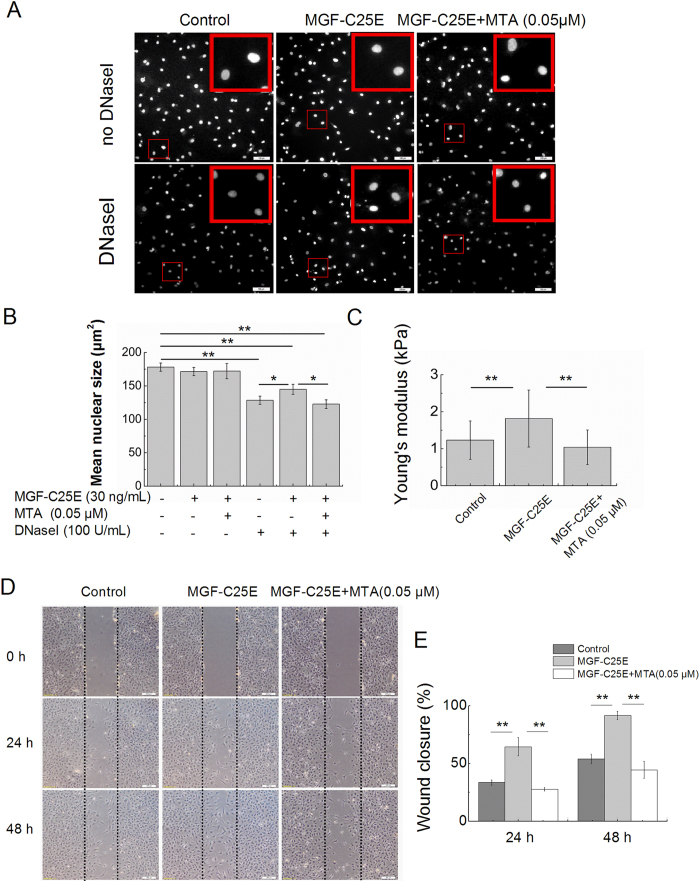
Effect of DNA methylation inhibitor (MTA) on MGF-C25E-induced chromatin condensation, nuclear stiffness and cell migration. (**A**) *In situ* DNaseI-sensitivity assay. Tenocytes were exposed to MGF-C25E (30 ng/mL) in the absence or the presence of MTA (0.05 μM) for 24 h. DAPI-stained nuclei after 20 minutes of digestion with the 100 U/mL of DNaseI are shown. Bars = 100 μm. (**B**) Quantitative analysis of the mean size of nucleus after DNaseI exposure as shown in A. n = 3 in each group. (**C**) The nuclei of tenocytes exposed to MGF-C25E (30 ng/mL) show a higher Young’s modulus than those of the control cells, whereas pretreatment with MTA (0.05 μM) for 60 min inhibited the increase of Young’s modulus in tenocytes induced by MGF-C25E. n = 20 in each group. (**D**) Images of the migration of tenocytes treated with MTA, as evaluated through a scratch wound assay. Bars = 200 μm. The migration was evaluated by determining the ratio of the residual wound area to the initial wound area. n = 3 in each group. Data are expressed as the means ± SD; *p < 0.05 and **p < 0.01.

To further examine the role of MGF-C25E-induced DNA methylation in chromatin condensation, in nuclear stiffness, and in migration of tenocytes, DNA methylation was inhibited using the methylase inhibitor 5’-deoxy-5’-methylthioadenosine (MTA). MTA (0.05 μM) suppressed the elevation in the DNA methylation levels induced by MGF-C25E (Fig. 7A,B). MTT analysis showed that MTA at a concentration of 0.05 μM had no effect on tenocyte viability (Fig. 7C). Furthermore, inhibition of DNA methylation also inhibited MGF-C25E-induced chromatin condensation (Fig. 8A,B). AFM assays showed that blockade of DNA methylation abolished the MGF-C25E-promoted nuclear stiffness (Fig. 8C). Wound healing assays further showed that blockade of DNA methylation abrogated the MGF-C25E-promoted migration of tenocytes (Fig. 8D,E). These results demonstrate that MGF-C25E-induced DNA methylation plays a crucial role in regulating chromatin condensation, nuclear stiffness, and migration in tenocytes.

## Discussion

We have previously demonstrated that MGF-C25E promotes the migratory potential of *in vitro* cultured tenocytes via the activation of the FAK-ERK1/2 signaling pathway. In this study, we report that MGF-C25E promotes tenocyte migration by inducing DNA methylation and by increasing chromatin condensation and nuclear stiffness via the FAK-ERK1/2 signaling pathway. This new finding is a close link for MGF-C25E signal transduction from the cytoplasm into the nucleus of tenocytes, and it uncovers the important roles of nuclear mechanics in MGF-C25E-promoted tenocyte migration.

At present, there is disagreement as to the effect of nuclear stiffness on cell migration. Gerlitz and Bustin hold that the cells with stiffer nuclei are easier to move^16^,^23^. The stiffness of the nucleus may affect the outcome of forces applied to it by the cytoskeleton. Forces applied to a highly elastic nucleus would be dispersed into many directions, making it harder to push the nucleus and control its migration towards a specific cellular location. Conversely, forces applied to a stiffer nucleus would stay more focused, making it easier to regulate its shape and direction of migration^16^. However, some studies suggest that a more deformable nucleus may confer a significant advantage for cell migration^26^,^27^. Remarkably large nuclear deformations are observed during the migration of stem cell-like progenitor cells in brain tissue^28^. Discher *et al.* considered that stem cell nuclei, which are more plastic than those of fully differentiated cells, are highly contractile and can generate significant cytoskeletal stress, representing a potential driving force for cell motility^27^,^29^. In our study, using a scratch wound assay, we found that MGF-C25E can promote tenocyte migration by increasing nuclear stiffness in 2D surfaces. This result agrees with Gerlitz and Bustin’s finding^24^, suggesting that a stiffer nucleus is better for a cell to migrate in 2D surfaces.

The nuclear lamina is usually considered to be a major contributor to the mechanical properties of the nucleus^30^. In our study, the MGF-C25E clearly increases nuclear stiffness; however, the surprising finding of this study is that there is no significant change in Lamin A/C expression in MGF-C25E-treated tenocytes, suggesting that MGF-C25E-increased nuclear stiffness is independent of Lamin A/C. The nuclear lamina assembles underneath the nuclear envelope and consists of A-type lamins (mainly lamins A and C), B-type lamins (lamins B1 and B2 in somatic cells), and lamin-associated membrane proteins, which connect lamins to both intranuclear chromatin and the cytoskeleton^31^. B type lamins are essential for viability but have no effect on nuclear stiffness^30^; A-type lamins form thick layers that provide rigidity^27^,^32^,^33^. However, Discher *et al.* demonstrated that the stiffness of the nuclear lamina is a barrier to 3D migration, but does not affect 2D migration^27^. Moreover, the stiffness of the nucleus is defined by a number of factors, such as the nuclear membranes, nuclear lamina and chromatin structure^23^,^32^,^34^. Discher *et al.* demonstrated that changes in the global condensation level of chromatin have a significantly larger effect on stiffness of the nucleus than changes in Lamin A/C^28^. In our study, MGF-C25E did not affect Lamin A/C expression, but it clearly promoted chromatin condensation, indicating the key contribution of MGF-C25E-promoted chromatin condensation to nuclear stiffness.

In the mammalian genome, DNA methylation is an epigenetic mechanism that involves a change of chromatin structure, DNA conformation, DNA stability and the mode of interaction between DNA and protein^35^. There has been recent interest in characterizing links between DNA methylation and chromatin condensation. Gerlitz and Bustin have proven that DNA methylation promotes chromatin conformation in murine B16-F1 cells^23^. In our study, we found that MGF-C25E promotes DNA methylation. We also found that inhibition of DNA methylation inhibits the elevation of chromatin condensation induced by MGF-C25E. Moreover, AFM assays showed that blockade of DNA methylation inhibits the MGF-C25E-increased nuclear stiffness. Our study provides evidence of a link for DNA methylation to chromatin condensation and nuclear mechanics.

The FAK-ERK1/2 signaling pathway plays an important role in numerous biological behaviors of a cell. ERK1/2 is a member of the family of mitogen-activated protein kinases (MAPK) that are activated in response to the signals originating from integrins and growth factor receptors. ERK1/2 activation has been implicated as a regulatory component in cell motility^36^,^37^. Ficz *et al.* found that inhibition of ERK1/2 signaling using small-molecule inhibitors in murine embryonic stem cells (ESCs) induced hypomethylation^38^. Moreover, other studies showed that DNA methyltransferases (Dnmts) could be regulated by the ERK1/2 signal^39^,^40^. DNA methylation is catalyzed by a family of Dnmts that transfer a methyl group from S-adenosyl methionine (SAM) to the fifth carbon of a cytosine residue to form 5mC^35^. FAK is a focal adhesion-associated protein kinase involved in cellular adhesion and spreading processes. Cells lacking the tyrosine kinase FAK have larger and more numerous adhesions and migrate poorly^41^. Although there is no direct study showing that the FAK signal can regulate DNA methylation or Dnmts, ERK1/2 is a well-known downstream effector of FAK, and some studies have shown that the FAK signal plays a role in cells through the ERK1/2 signal^42^,^43^. Moreover, FAK is a primary signaling mediator of dynamic changes in actin cytoskeletal reorganization^44^. Nuclear shaping by the cytoskeleton has been described in several systems^45^,^46^. We previously demonstrated that the FAK-ERK1/2 signaling pathway is necessary for MGF-C25E-promoted tenocyte migration. In the current study, we prove that MGF-C25E induces DNA methylation, increases chromatin condensation, enhances nuclear stiffness, and promotes migration in tenocytes via the FAK-ERK1/2 signaling pathway. These findings clarify a mechanism for MGF-C25E signal transduction from the cytoplasm into the nucleus in MGF-C25E-promoted tenocyte migration.

## Conclusion

In this study, we have demonstrated that MGF-C25E promotes tenocyte migration by increasing nuclear stiffness through DNA methylation and chromatin condensation via the FAK-ERK1/2 signaling pathway. Our results identify the molecular mechanisms involved in MGF-C25E-triggered cellular signaling in tenocyte migration, and also provide strong evidence for the role of nuclear mechanics during cell migration. These findings are helpful towards a better understanding of the role of MGF in tendon wound healing, and they may serve as the basis for a potential therapeutic strategy in tissue repair and regeneration of the injured tendon.

## Materials and Methods

### Primary culture of rat tenocytes

Male Sprague-Dawley rats (Laboratory Animal Center, the Third Military Medical University, China) weighing 150–200 g were used as the source for tenocytes in this study. All of the procedures were approved by the Chongqing Science and Technology Commission, Chongqing, China. The methods were carried out in accordance with the approved guidelines. The tenocytes were harvested from the Achilles tendons of rats through aseptic procedures. Briefly, each tendon tissue was cut into pieces that were approximately 1.5–2.0 mm^3^ in volume, which were separately placed into culture flasks. Then, 3 mL of Dulbecco’s modified Eagle’s medium (DMEM) supplemented with 10% fetal bovine serum (FBS, Hyclone, Logan, UT, USA), 100 U/mL penicillin, and 100 μg/mL streptomycin was added to the flasks. The culture flask was then placed upside down in an incubator with 95% air and 5% CO_2_ at 37 °C for 12 h to promote the attachment of the tissues and was then inverted. After the tenocytes migrated from the explants, the culture medium was replaced every three days. After reaching 80–85% confluence, the tenocytes were subcultured with 0.25% trypsin-0.02% EDTA at a dilution rate of 1:2. Cells from passages 2 or 3 were used for the experiments.

### Scratch wound migration assay

The migration was assessed through a wound healing assay. The tenocytes were seeded at a density of 4 × 10^4^ cells/well in a 24-well culture plate. After reaching 80–85% confluence, the tenocytes were synchronized by serum starvation for 12 h. The confluent monolayer was then scratched with a 10-μL pipet tip and washed twice with PBS. The wells were filled with 1 mL of serum-free DMEM with or without MGF-C25E (Phoenix Pharmaceuticals, Burlingame, USA). To test whether chromatin decompaction inhibited the rate of cell migration, we examined the effect of the levels of DNA methylation on the rate of cell migration. The cells were incubated with histone deacetylase (HDAC) inhibitor trichostatin A (TSA) (Beyotime, Jiangsu, China) or methylase inhibitor 5’-deoxy-5’-methylthioadenosine (MTA) (Sigma-Aldrich, St. Louis, MO, USA) for 60 min at 37 °C before scratching. Images of the wounds were acquired at 0, 24, and 48 h through a microscope (Olympus, Japan). Using Image J, the levels of wound closure were assessed by calculating the ratio of the closure area to the initial wound area as follows: 
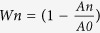
 × 100 [%], where *Wn* represents the percentage of wound closure, *An* represents the residual wound area at the metering time point (h), and *A0* represents the initial wound area.

### Isolation of the nucleus

Nuclei were isolated from the tenocytes as previously reported^47^. Briefly, confluent cells were washed with PBS (Dulbecco’s phosphate-buffered saline without Ca^2+^ or Mg^2+^, Sigma-Aldrich, St. Louis, MO, USA) and treated with an ice-cold low-ionic-strength extraction solution consisting of 2.5 mM triethanolamine (Sigma-Aldrich, St. Louis, MO, USA), 1 mg/mL leupeptin (Sigma-Aldrich, St. Louis, MO, USA), and 1 mg/mL pepstatin (Sigma-Aldrich, St. Louis, MO, USA) in distilled water for 10 min. The cells were then treated with 0.05% NP-40 in PBS (hereafter, containing 1 mg/mL leupeptin and 1 mg/mL pepstatin and kept at 4 °C) for 1 min. The dorsal side of many cells was broken away, and the nuclei were popped out by shaking the dish gently under a phase-contrast microscope. A small volume (approximately 1 mL) of the supernatants containing the isolated nuclei was aspirated with a pipette and suspended in 10 mL PBS. The solution was centrifuged at 400 × g for 10 min at 4 °C with two changes of PBS. The isolated nuclei were seeded on sterilized 24-mm coverslips coated with poly D-lysine (Sigma-Aldrich, St. Louis, MO, USA) in a six-well culture plate. The culture plate was then placed in an incubator with 95% air and 5% CO_2_ at 37 °C for 3 h to favor the attachment of nucleus.

### Atomic force microscopy (AFM) analysis

The stiffness of isolated nuclei was measured using an AFM (JPK, Berlin, Germany) mounted on an inverted microscope (Leica, Solms, Germany) at 37 °C. A soft silicon nitride quadratic pyramid tip (0.02 N/m) was used at an angle of 17.5°. A single nucleus with normal morphology was identified using an optical microscope, and the AFM cantilever probe was positioned on the nucleus region. The cantilever was descended toward the nucleus at a ramp speed of 3 μm/s. The force-distance curves were collected and analyzed using the JPK Imaging Process Software to obtain the Young’s modulus (*E*).

### RNA preparation and Real-time Polymerase Chain Reaction (qRT-PCR)

Total RNA was isolated from tenocytes using the RNeasy minikit (BioTeke, Beijing, China) according to the instructions of the manufacturer. A 500-ng aliquot of each sample of total RNA was reverse-transcribed in 20 μL using Reverse Transcriptase kit (TaKaRa, Japan). Levels of Lamin A/C gene were determined by real-time PCR (qRT-PCR) performed with *CFX96* Touch™ *Real-Time PCR* system (Bio-Rad, Hercules, CA, USA) using a reagent kit (TaKaRa, Japan) containing the fluorescence dye SYBR green and the reference dye ROX. GAPDH was used as a reference gene. Amplification of cDNA samples was carried out according to the following protocol: initial denaturation at 95 °C for 30 s; denaturation at 95 °C for 5 s, annealing at 58 °C for 40 s, 40 cycles. An additional step, a melting curve, was added to determine specificity which was carried out according to the following protocol: dissociation at 95 °C for 15 s, 65 °C for 1 min, and 95 °C for 15 s. The following PCR primers were used: rat Lamin A/C (NM_001002016), forward, 5’-TGGTTGAGGACAATGACGATGA-3, reverse, 5’-GGTGCTGACGGCAGGTTGTA-3’; rat GAPDH (NM_017008), forward, 5’-AAGTTCAACGGCACAGTCAAGG-3’, reverse, 5’-CGCCAGTAGACTCCACGACATA-3’. The results were analyzed using CFX Manager Software (Bio-Rad, Hercules, CA, USA).

### SDS-polyacrylamide gel electrophoresis (PAGE) and western blot analysis

The tenocytes were seeded at a density of 5 × 10^4^ cells/well in a six-well culture plate. After reaching 80–85% confluence, the cells were subjected to serum starvation for 12 h and then treated with FBS-free DMEM containing 30 ng/mL MGF-C25E for 24 h. To block the FAK or ERK1/2 signals, the cells were incubated with 10 μM FAK inhibitor PF573228 (Tocris Bioscience, R&D Systems, Minneapolis, USA) or 50 μM ERK1/2 inhibitor PD98059 (Beyotime, Jiangsu, China) for 30 min at 37 °C before treatment with MGF-C25E. The cells were washed with ice-cold PBS and digested immediately after exposure to MGF-C25E for 24 h. The proteins were extracted using cell lysis buffer. After electrophoretic separation by 8% SDS-polyacrylamide gel electrophoresis, the proteins were electrotransferred onto polyvinylidene fluoride (PVDF) membranes (Millipore, Billerica, MA, USA). The membranes were then blocked with Tris-buffered saline containing 0.1% Tween-20 (TBST) and 5% skim milk for 1 h at room temperature. Lamin A/C mouse mAb (Cell Signaling Technology, Danvers, MA, USA), and β-actin rabbit mAb (4A Biotech, Beijing, China) were used according to the manufacturer’s protocols. The membranes were incubated overnight with these antibodies at 4 °C with slight shaking. The membranes were then washed three times in TBST and further incubated with an HRP-conjugated antibody (Beyotime Biotech, Shanghai, China) for 1 h at room temperature. After three washes in TBST, the signals were developed using an enhanced chemiluminescence kit (KeyGEN Biotech, Nanjing, China). A semiquantitative evaluation of the bands was performed by densitometry (VersaDoc, Bio-Rad, Hercules, CA, USA). The levels of Lamin A/C proteins were determined by normalizing to the protein level of β-actin.

### Immunostaining

Tenocytes were seeded on confocal dishes. After reaching 80–85% confluence, the cells were synchronized by serum starvation for 12 h. The tenocytes were then treated with serum-free DMEM with or without 30 ng/mL MGF-C25E for 24 h. To block the FAK or ERK1/2 signals, the tenocytes were incubated with the inhibitor of FAK (10 μM PD98059) or the inhibitor of ERK1/2 (50 μM PD98059) for 30 min at 37 °C before treatment with MGF-C25E. To block DNA methylation, the tenocytes were incubated with MTA for 60 min at 37 °C before treatment with MGF-C25E. The cells were then washed with ice-cold PBS and fixed in 4% paraformaldehyde-PBS at room temperature for 30 min. Lamin A/C mouse mAb (Cell Signaling Technology, Danvers, MA, USA) and 5-methylcytosine (5-mC) rabbit mAb (Abcam, Cambridge, MA, USA) were used according to the manufacturer’s protocols, and the cells were incubated overnight with these antibodies at 4 °C with shaking. The cells were then washed three times in PBST and further incubated with an anti-mouse Cy3-conjugated antibody or anti-rabbit Cy3-conjugated antibody (Beyotime Biotech, Shanghai, China) for 1 h at room temperature. After three washes in PBST, the nuclei were labeled with DAPI (Sigma-Aldrich, St. Louis, MO, USA) for 10 min at room temperature. The cells were photographed using a fluorescence microscope (Olympus, Japan), and the intensities of the mean fluorescent signals were measured using Image J software.

### *In situ* DNaseI-sensitivity assay

The tenocytes were seeded at a density of 4 × 10^4^ cells/well in a 24-well culture plate. After reaching 80–85% confluence, the cells were synchronized by serum starvation for 12 h and treated with serum-free DMEM with or without MGF-C25E for 24 h. The cells were then washed once with PBS and lysed in CSK buffer supplemented with 0.2% Triton X-100 and protease inhibitor cocktail (Sigma-Aldrich, St. Louis, MO, USA) at room temperature for 5 minutes. Next, the samples were incubated in CSK buffer supplemented with 0.1% Triton X-100, protease inhibitor cocktail and DNaseI (Sigma-Aldrich, St. Louis, MO, USA) at the indicated concentrations at room temperature for 20 minutes. The remaining DNA was stained using DAPI at a concentration of 1 μg/mL in CSK buffer supplemented with 125 mM ammonium sulfate and protease inhibitor cocktail for 10 minutes. Following fixation in methanol at –20 °C for 5 minutes, the cells were washed in CSK buffer and photographed using a fluorescence microscope (Olympus, Japan). The area of the nucleus was measured using Image J software.

### MTT assay

The MTT (3-(4,5-dimethylthiazol-2-yl)-2,5-diphenyl-tetrazolium bromide) assay was performed to measure the proliferation of tenocytes. Tenocytes were seeded at a density of 3 × 10^3^ cells/well in a 96-well culture plate. After adherence, tenocytes were serum-starved overnight and then treated with MTA. Then, 5 mg/mL MTT reagent (Sigma-Aldrich, St. Louis, MO, USA) was added into each well at the indicated treatment time. After incubation at 37 °C for 4 h, the media was removed and the formazan crystals in the cells were solubilized with 200 μL of dimethyl sulfoxide (DMSO). The formazan was then quantified using a microplate reader (Model 680, Bio-Rad, Hercules, CA, USA).

### Statistical analysis

The results were analyzed statistically using Student’s t-test and analysis of variance. Bonferroni post-hoc tests were used when the *p* value indicated a significant difference between the groups. The data are expressed as the means ± SD. *p *< 0.05 was deemed to be statistically significant.

## Additional Information

**How to cite this article**: Zhang, B. *et al.* Increased nuclear stiffness via FAK-ERK1/2 signaling is necessary for synthetic mechano-growth factor E peptide-induced tenocyte migration. *Sci. Rep.* **6**, 18809; doi: 10.1038/srep18809 (2016).

## Figures and Tables

**Figure 1:**
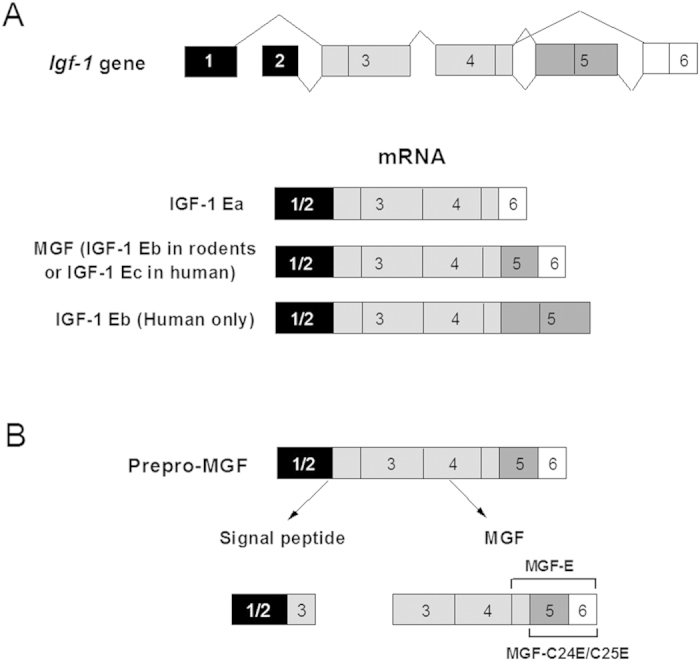
The generation of MGF by alternative splicing of the *igf-1*gene. (**A**) The *igf-1* gene has six exons. Exons 1 and exon 2 (black) serve as leader exons, either of which can be spliced to exon 3 and exon 4 (light gray). The mRNA variants with exon 4 spliced to exon 6 (white) are designated as IGF-1 Ea, while those variants with exon 4 spliced to exon 5 (gray) and exon 6 are designated as IGF-1 Eb in rat or IGF-1 Ec in human. The exon 4 spliced directly to exon 5 is designated as IGF-1 Eb, which exists only in humans. (**B**) The translation of MGF mRNA sequence generates prepro-MGF. This is followed by the removal of the signal peptide and the liberation of MGF. A 49-bp insert of exon 5 in MGF mRNA results in the distinct E-domains from the other variants.
